# Oligonucleotide and Parylene Surface Coating of Polystyrene and ePTFE for Improved Endothelial Cell Attachment and Hemocompatibility

**DOI:** 10.1155/2012/397813

**Published:** 2012-03-12

**Authors:** Martina Schleicher, Jan Hansmann, Bentsian Elkin, Petra J. Kluger, Simone Liebscher, Agnes J. T. Huber, Olaf Fritze, Christine Schille, Michaela Müller, Katja Schenke-Layland, Martina Seifert, Heike Walles, Hans-Peter Wendel, Ulrich A. Stock

**Affiliations:** ^1^Department of Thoracic, Cardiac and Vascular Surgery, University Hospital, Hoppe-Seyler-Strasse 3, 72076 Tuebingen, Germany; ^2^Fraunhofer Institute for Interfacial Engineering and Biotechnology, Nobelstrasse 12, 70569 Stuttgart, Germany; ^3^Department of Prosthodontics, Section of Medical Materials and Technology, University Hospital, Osianderstrasse 2-8, 72076 Tübingen, Germany; ^4^Inter-University Centre for Medical Technology (IZST), Eberhard Karls University, Silcherstrasse 7, 72076 Tuebingen, Germany; ^5^Institute of Medical Immunology, Charité Universitätsmedizin Berlin, Föhrer Straße 15, 13353 Berlin, Germany; ^6^Berlin-Brandenburg Center for Regenerative Therapies (BCRT), Charité Universitätsmedizin Berlin, Föhrer Straße 15, 13353 Berlin, Germany

## Abstract

*In vivo* self-endothelialization by endothelial cell adhesion on cardiovascular implants is highly desirable. DNA-oligonucleotides are an intriguing coating material with nonimmunogenic characteristics and the feasibility of easy and rapid chemical fabrication. The objective of this study was the creation of cell adhesive DNA-oligonucleotide coatings on vascular implant surfaces. DNA-oligonucleotides immobilized by adsorption on parylene (poly(monoaminomethyl-para-xylene)) coated polystyrene and ePTFE were resistant to high shear stress (9.5 N/m^2^) and human blood serum for up to 96 h. Adhesion of murine endothelial progenitor cells, HUVECs and endothelial cells from human adult saphenous veins as well as viability over a period of 14 days of HUVECs on oligonucleotide coated samples under dynamic culture conditions was significantly enhanced (*P* < 0.05). Oligonucleotide-coated surfaces revealed low thrombogenicity and excellent hemocompatibility after incubation with human blood. These properties suggest the suitability of immobilization of DNA-oligonucleotides for biofunctionalization of blood vessel substitutes for improved *in vivo* endothelialization.

## 1. Introduction

Current blood vessel replacement concepts, which use either prosthetic or biological grafts, achieve excellent mid-term results. The clinical application, however, is accompanied by a variety of limitations. Biological conduits (e.g., autologous greater saphenous vein) have better hemodynamic characteristics and avoid long-term anticoagulation but are limited in availability. On the long term, however, they fail due to intimal hyperplasia and fibrosis. Allograft transplants such as superficial femoral arteries have optimal hemodynamic properties, avoid any anticoagulation, and are resistant to infections to a certain extent. However, due to tissue scarcity, their availability is limited. In general, 5–30% of patients have no suitable autologous grafts available due to previous use or concomitant disease. In these cases, expanded polytetrafluoroethylene (ePTFE) grafts are used. However, despite their prevalence, these grafts have a lower patency rate due to noncompliance, thrombogenic surface, and the tendency to form intimal hyperplasia in particular in anastomotic areas [1]. None of the currently available blood vessel substitutes possess any regenerative or growth potential. This shortcoming is crucial especially for the treatment of pediatric patients and grafts with repeated injury (e.g., hemodialysis shunts).

The multidisciplinary approach of tissue engineering might offer an attractive pathway to overcome these shortcomings and develop blood vessel substitutes identical or similar to native human arteries. In vascular grafts, an *in vitro* established autologous endothelial cell layer on the implant surface prior to implantation provides an excellent barrier between the synthetic material and the blood flow and has been reported to reduce thrombosis and intimal hyperplasia [2–4]. Overcoming these limitations will result in a distinct improvement of graft performance and patency [2–5]. Indeed, clinical studies have demonstrated that such grafts can function as well as autologous vein grafts [6]. However, this concept imposes a variety of demands for successful transfer from the preclinical large animal setups to the clinical arena. Due to the high risk of bacterial and fungal infection throughout the entire *in vitro* culture (which takes up to 6 weeks from cell harvest to implantation of the engineered product), the process requires a cost-intensive infrastructure.

Accordingly, ongoing research focuses on the development of facilitated spontaneous *in vivo* endothelialization of vascular implants [7–11]. Several pathways are conceivable to achieve endothelialization *in vivo*. One is the capture, immobilization and adhesion of circulating endothelial (EC) or endothelial progenitor cells (EPC) from the blood stream. An alternative is promotion of transanastomotic ingrowth of endothelial cells from the native vessel on and into the graft. Both require a suitable graft surface for cell adhesion and proliferation. However, it is reported that EC do not adhere to currently available vascular graft materials like ePTFE and polyethylene terephtalate (Dacron) [12, 13]. Furthermore, transanastomotic ingrowth of EC in currently used vascular grafts does not exceed 1-2 cm even after years of clinical implantation [14]. Hence, materials that promote *in situ* endothelialization of cardiovascular implants (without intimal hyperplasia or thrombus formation during endothelium development) would be highly desirable. Different approaches exist, mostly using extracellular-matrix- (ECM-) derived proteins or peptides with functional domains of ECM proteins [7]. 

Another intriguing coating material for implant surfaces is deoxyribonucleic acid (DNA). DNA is a naturally occurring material with a homogenous molecular structure in all vertebrate species [15, 16]. DNA is nontoxic with little or no immunogenicity in contrast to other biological antigens like proteins and sugars [15, 17, 18]. Another advantage of DNA molecules are their easy and rapid chemically synthetization. Use of DNA as coating material has already been suggested for dental applications [19], and a DNA-chitosan complex was used as scaffold material for tissue engineering [20]. Van Den Beucken et al. fabricated multilayered DNA coatings consisting of poly-D-lysine (PDL) or poly(allylamine hydrochloride) (PAH) and DNA and demonstrated increased proliferation of primary rat dermal fibroblasts (RDFs) and cyto- as well as histocompatibility of the multilayered DNA coatings [21]. Challenges in application of DNA for surface functionalization are the easy nucleolytic degradation of the substance and its solubility in aqueous solutions [19]. Furthermore, DNA is an optimal material for immobilization of additional growth or adhesion factors, as DNA offers accessible functional groups for chemical coupling reactions and the possibility to incorporate molecules via groove binding and intercalation [22, 23]. 

Apart from controlled cell adhesion, the coating matrix needs to possess excellent mechanical properties in terms of flexibility and long-lasting *in vivo* adherence to the implant surface. These properties are known to be supplied by poly (p-xylylene) (PPX) and its derivates, known by their commercial trade name as parylenes [24, 25]. Parylenes can be conformally coated onto irregular substrates by a vapor deposition process and are chemically inert and nonbiodegradable. The FDA has already recognized some parylenes, such as Parylene C and Parylene N, as Class VI polymers for coating implanted medical devices due to their biocompatibility [26, 27].

Here, we report a concept of a stable DNA coating and EC and EPC adhesion on DNA coatings using continuous shear stress application.

## 2. Materials and Methods

### 2.1. Oligonucleotides

Oligonucleotides were synthesized by TIB Molbiol (Berlin, Germany). As single-sequence 5′-GGGAGCTCAGAATAAACGCTCAACAACCCGTCAACGAACCGGAGTGTGGCAGGTTCGACATGAGGCCCGGATC-3′ was used. The DNA oligonucleotide library contained a 40-base central random sequence: 5′- GAATTCAGTCGGACAGCG-N_40_-GATGGACGAATATCGTCTCCC-3′. For detection, oligonucleotides were 5′-labeled with 6-carboxyfluorescein (6FAM). For immobilization, a 5′-C12-NH_2_ modification was used.

### 2.2. Cells and Culture

Murine embryonic EPC (eEPC) line T17b cells [28] were cultured in flasks precoated with 0.1% gelatin. The culture medium consisted of DMEM (4.5 g/l glucose, Lonza, Köln, Germany) supplemented with 20% FCS, 0.1 mmol/l *β*-mercaptoethanol (Serva, Heidelberg, Germany), 1 mmol/l nonessential amino acids (Gibco, Paisley, UK), 100 U/mL penicillin, 100 *μ*g/mL streptomycin (Gibco, Paisley, UK), and 2 mmol/l L-glutamine (Lonza, Köln, Germany).

Human umbilical vein endothelial cells (HUVECs) were cultured in VascuLife VEGF Culture Medium containing 2% FCS (Lifeline Cell Technology, Walkersville, USA).

Human ECs were harvested from human saphenous veins. 4 to 5 cm long remnants of saphenous veins from patients undergoing aortocoronary bypass procedures were used as a cell source. Tissue procurement and use were approved by our local ethics committee and informed consent was obtained. The veins were cannulated, flushed with DMEM (4.5 g/L glucose, Lonza, Köln, Germany), and filled with 0.2% collagenase A (Roche, Mannheim, Germany). Cells were harvested after 20 min of collagenase incubation at 37°C, 5% CO_2_. The culture medium consisted of DMEM (4.5 g/l glucose, Lonza, Köln, Germany) supplemented with recombinant basic fibroblast growth factor (bFGF, 10 ng/mL; Boehringer, Ingelheim, Germany), 100 U/mL penicillin, 100 *μ*g/mL streptomycin (Gibco, Paisley, UK), and 10% FCS.

### 2.3. Parylene Deposition Process

Standard ePTFE vascular prostheses were obtained from Jotec GmbH, Hechingen, Germany (FlowLine Bipore and FlowLine Bipore Heparin), and ePTFE patch material from W.L. Gore & Associates, Inc., Flagstaff, Arizona, USA (Gore-Tex Cardiovascular Patch). ePTFE was activated using microwave plasma (400 W, hydrogen and nitrogen in equal parts, 30 s) prior to coating with parylenes. Poly (monochloro-para-xylene) (“Galxyl C”-parylene C, from Galentis S.P.A., Marcon, Italy) and poly (monoaminomethyl-para-xylene) (diX AM-aminoparylene, from Kisco Conformal Coating Ltd., Düsseldorf, Germany) were coated on polystyrene dishes, polypropylene sheets, and activated ePTFE using a chemical vapour deposition (CVD) process in a PDS 2010 coater (SCS Specialty Coating Systems, Indiana, USA). A general description of parylene CVD processes is given by Lahann [29]. This technique enables thin homogeneous and conformal deposition on a variety of substrates. Deposition is performed under vacuum at room temperature. A thin (ca. 0.1 *μ*m) diX AM coating was deposited onto a thicker (ca. 2 *μ*m) intermediate layer of inert Parylene C according to recommendations from the diX AM manufacturer. After deposition, the samples were stored in the dark using argon atmosphere to avoid amine's oxidation.

### 2.4. Surface Characterization

#### 2.4.1. Parylene Thickness Determination

The thickness of the intermediate Parylene C layer was determined by both gravimetry and ellipsometry on reference samples. In the first case, the mass increase of an aluminum foil which was parylene coated in the same deposition run was determined. For ellipsometry, single crystalline Si substrates were used. The thickness of diX AM parylene top coatings was determined by ellipsometry only. The ellipsometric measurements were performed on a SE 801 spectroscopic ellipsometer (Sentech Instruments GmbH, Berlin, Germany).

#### 2.4.2. XPS Measurements

The samples were stored in dark under argon and transferred into the XPS apparatus within 24 h of deposition. The surface elemental composition was verified by X-ray photoelectron spectroscopy (XPS) analysis using an Axis Nova system (Kratos Analytical, Manchester, UK) with monochromatic Al K*α* X-Ray source. For all samples, overview spectra and detailed spectra of all elements found in the overview were recorded.

#### 2.4.3. ESR Measurements

For determination of the number of chemically available amino groups on the diX AM surface electron spin resonance spectroscopy (ESR) in combination with chemical derivatization was applied (similar technique was used by Samal et al. [30] for qualitative detection of surface functionalization). First, functionalized stable free radicals (4-carboxy-TEMPO, Sigma-Aldrich) were bound to surface amino groups via an EDC-mediated reaction. Subsequently, the number of radicals in the sample was measured using ESR. Polypropylene film samples (45 × 90 mm) coated on both sides with diX AM were incubated for 24 hours with 0.65 mmL/l 4-carboxy-TEMPO, 1.55 mg/mL EDC in 0.1 M MES buffer (pH 5.0) at room temperature. After a thorough washing, the films were dried, tightly rolled, and then put into the sample holder tube of the ESR device (MS200, Magnettech GmbH). To obtain the absolute number of spins in the sample, the double integral of the measured signal was related to that of a Cr(III) standard. The number of spins measured should then equal the number of amino groups having reacted with the carboxy moiety of the 4-carboxy-TEMPO.

#### 2.4.4. Contact Angle Measurements

Contact angle measurements were performed to determine the changes in wettability of the prepared surfaces. The captive bubble technique was chosen for analyses because the surfaces were constantly in contact with fluid media. The coated samples were completely immersed in PBS with the coated side face down. The captive bubble contact angle measurements were taken at 22°C using a video capture system (OCA 40, DataPhysics Instruments GmbH, Filderstadt, Germany). An air bubble was brought in contact with the solid sample from below. After a few seconds, the static contact angle near the three-phase line was measured. For each sample, at least six contact angles were measured.

### 2.5. Oligonucelotide Immobilization on diX AM-Coated Surfaces

If not stated differently, 1.7 *μ*M DNA-oligonucleotides were applied to the diX AM-coated surface in HEPES buffer (20 mM, pH 7.0) at room temperature. After 2 h, Tris-HCl was added to a concentration of 50 mM and incubated for 15 min. For experiments with cell incubation, the surfaces were washed with 0.2% BSA. Then, a triple washing step with PBS followed. For cell experiments, an incubation with a PBS-antibiotic solution (10 mL PBS with 0.5 mL antibiotic-solution: 0.3 mg amikacin, 0.75 mg flucytosine, 0.3 mg vancomycin, 0.075 mg ciprofloxacin, 0.3 mg metronidazole) for 24 h at 4°C was performed. 

### 2.6. Shear Stress Resistance Test

**Figure 1:**
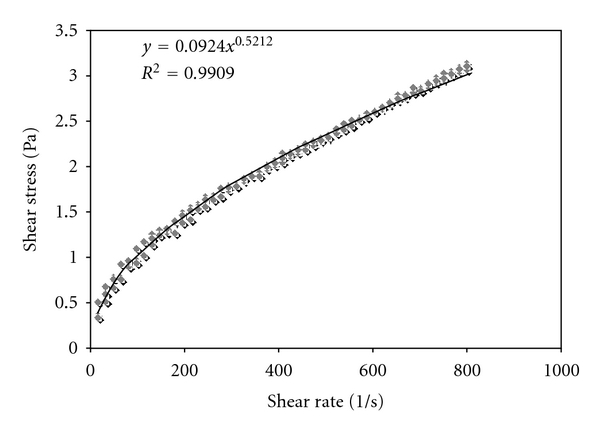
Shear stress dependency on shear rate of 1000 ppm Xanthan gum measured by rotational rheometry.

DiX AM-coated polypropylene sheets with applied oligonucleotides (0.5 *μ*M) were exposed to flow-induced shear stress for 1 h at 37°C in a closed circular system. The system consisted of silicon tubing, a fluid reservoir, a peristaltic pump, and a following vessel to attenuate pump-induced pressure variability. The test fluid consisted of 1000 ppm xanthan gum. Wall shear stress for 1000 ppm Xanthan gum, a non-Newtonian power-law fluid [31], was calculated as follows. For a non-Newtonian power-law fluid applies

(1) $$\tau_{zr}=k\gamma^{n},$$

where *τ*_*zr*_ is the shear stress in a tube at length *z* and radius *r* and *γ* the shear rate [32]. Shear-rate-dependent shear stress of the xanthan gum solution was determined by rotational rheometry (UDS 200, Physica, Ostflidern) using the following settings: shear rate 0.01–800 1/s, 50 measurement points, measurement duration per point 10 s, three individual 1000 ppm Xanthan gum solutions. From the measurement, the following values were gained: *k* = 0.0924 Pa ∗ *s* ^*n*^ and *n* = 0.5212 (Figure 1). To achieve a wall shear stress of *τ*_wall_ = 9.5 N/m^2^ in a tube with a radius of *R* = 0.135 cm, the required flow rate *Q* was calculated as follows [32]:

(2) $$Q=\frac{\pi R^{3}}{1/n+3}{\left(\frac{\tau_{\text{wall}}}{k}\right)}^{1/n}=\;11.4\;{\text{cm}}^{3}/\text{s}.$$

### 2.7. Serum Stability

DiX AM coated with oligonucleotides (0.5 *μ*M) was incubated with serum derived from fresh human blood for 15 min, 24 h, 48 h, 72 h, and 96 hours at 37°C. Serum was changed every 24 hours. Amount of oligonucleotide remaining on diX AM surface was determined.

### 2.8. Oligonucleotide Detection

Oligonucleotides were modified with 6FAM and could be detected by a fluorescence microplate reader (Mithras LB 940, BertholdTech, Bad Wildbad, Germany). For confirmation of the results and better quantification, an ELISA system was developed, using an antifluorescein antibody (Molecular Probes, Cat# A-889, Eugene, USA) and an alkaline-phosphatase-conjugated secondary antibody (ZYMED Laboratories, Invitrogen, Cat# 65-6122, Carlsbad, USA). Incubation time was 2 h at room temperature each. As substrate Alkaline Phosphatase Yellow (pNPP) Liquid Substrate System for ELISA (Sigma) was used. 

### 2.9. Dynamic Cell Adhesion Test

Oligonucleotides were applied to marked areas or the whole surface of diX AM coated Petri-dishes or diX AM-coated 12-well plates. Petri-dishes were incubated with 0.05 ∗ 10^6^ cells for 1–2.5 h at 37°C, 5% CO_2_ in cell-corresponding or experiment-dependent medium or buffer on a rocking orbital shaker (The Belly Button, Stovall Life Science Inc. Greensboro, USA). Following medium exchange to remove nonadherent cells, Petri-dishes were inspected by phase-contrast microscopy (Zeiss Axio Observer Z1, Germany). Cells in images with the same area were counted. An alamarBlue assay (AbD Serotec, Oxford, UK) was performed after 22 h to determine the relative number of metabolically active adherent cells. 150 *μ*L alamarBlue reagent were added to the samples in 1.5 mL medium and incubated for four hours at 37°C and 5% CO_2_. Two times 100 *μ*L of each sample were pipetted into a clear flat bottom microplate and measured at 530 nm extinction and 600 nm emission wavelength (Mithras LB 940, BertholdTech, Bad Wildbad, Germany). 

### 2.10. Staining of Adhesion Markers

Oligonucleotides were applied to marked areas of diX AM coated Petri-dishes. Oligonucleotide-coated and diX AM-coated Petri-dishes and tissue-culture Petri-dishes were incubated with 0.05 ∗ 10^6^ HUVECs at 37°C, 5% CO_2_ in medium containing FCS on a rocking orbital shaker (The Belly Button, Stovall Life Science Inc, Greensboro, USA). After one hour medium including non-adherent cells was removed. Adherent cells were fixed with 4% paraformaldehyde (Sigma) for 10 min at room temperature. After a washing step with PBS (Lonza), PBS containing 2% FCS and 0.1% Triton (Sigma) was applied for 15 min. Samples were incubated over night at 4°C with the primary antibodies (anti-vinculin or CD49e (Integrin alpha 5), abcam, Cambridge, UK) in PBS containing 2% FCS and 0.1% Triton. After a washing step, the secondary antibody (Goat polyclonal Secondary Antibody to Mouse IgG-H&L (Chromeo 488), abcam, Cambridge, UK) was applied for 1 h at 37°C. Cells were stained with phalloidin (Alexa Fluor 568 phalloidin, Invitrogen, Carlsbad, USA) for 20 min at room temperature following a further washing step with PBS. As a last staining step, cells were incubated with DAPI (Sigma) for 2 min. Following staining of adherent cells, Petri-dishes were inspected by fluorescence microscopy at a magnification of 200x (Zeiss Axio Observer Z1, Germany).

### 2.11. Viability Test over a Period of 14 Days

For viablity tests, standard ePTFE vascular prostheses either uncoated or coated with heparin (FlowLine Bipore and FlowLine Bipore Heparin, Jotec GmbH, Hechingen, Germany) or ePTFE (FlowLine Bipore) coated with Parylene C, diX AM, and oligonucleotieds as described above were used. The three differently coated materials were cut into samples and glued with silicone paste into 48-well suspension culture plates. 3 ∗ 10^4^ HUVECs were seeded into each well. After 2.5 h, incubation using continuous agitation (The Belly Button, Stovall Life Science Inc, Greensboro, USA) medium was exchanged and nonadherent cells removed. Cell viability was measured with the alamarBlue assay after 1, 2, 5, 8, 12, and 14 days.

### 2.12. Hemocompatibility Testing

12-well Polystyrene plates and ePTFE patches (Gore-Tex Cardiovascular Patch, W.L. Gore & Associates, Inc., Flagstaff, Arizona, USA) were coated with diX AM and oligonucleotides as described above. ePTFE was cut into 12-well-sized samples and glued into 12-well suspension culture plates using silicone paste. Human blood samples were obtained from six healthy volunteers following giving informed consent (approved by the ethical committee of University Hospital Tuebingen). Whole blood from each volunteer was collected in heparin precoated monovettes (1,5 IU Liquemin, Roche, Grenzach-Whylen, Germany per mL blood). Two 12-wells in each group were incubated with 3 mL donor blood each. After 1 h incubation at 37°C with continuous shaking, the two blood samples of each group were pooled and further processed using the following protocols. 2.4 mL blood from each group was added to an EDTA-monovette. Blood cell count, including platelet count, was performed using a Cobas Micros 60 S/N (Axon Lab, Reichenbach, Germany). 2.8 mL blood from each group was collected in a Citrate-monovette. Monovettes were centrifuged at 2000 g for 15 min at room temperature. 600 *μ*L supernatant was used for thrombin-antithrombin complex (Enzygnost TAT micro, Dade Behring Marburg, Germany) and PMN-Elastase determination (Milenia PMN-Elastase, Milenia Biotec, Germany). 5.4 mL blood from each group was added to 0.3 mL of CTAD anticoagulant monovettes (Becton Dickinson, USA). Tubes were placed on ice for 15 min. Following centrifugation at 2500 g for 20 min at 4°C 1.4 mL, plasma from the middle fraction was aspirated and transferred to a new neutral monovette. After repeated centrifugation, 200 *μ*L plasma was used for determination of *β*-Thromboglobulin (ASSERACHROM *β*-TG Kit, Diagnostica Stago, France).

### 2.13. Statistics

All quantitative results were expressed as mean standard deviation. The differences among groups were analyzed with one-way ANOVA, and independent samples *t*-test was performed between each of two groups in case of a significant statistical difference existed. *P* values < 0.05 were considered statistically significant. 

## 3. Results

### 3.1. DiX AM and Oligonucleotide Coating Surface Characterization

Substrates were coated with parylene C as an intermediate layer and with diX AM as top coating. Coating thicknesses measured by ellipsometry on a simultaneously coated Si wafer were 550–2200 nm for parylene C and 40–120 nm for diX AM, depending on charge deposition parameters. The elemental composition in percent of total number of atoms excluding hydrogen of the surface measured by XPS was as follows: N-6.2 at.% (theoretically expected for diX AM-5.9 at.%), C-90.7 at.%, Cl-1.9 at.%, and O-1.2 at.%. Chlorine is not contained in diX AM but makes up 14.2 at.% of Parylene C, which was the intermediate layer under diX AM. Thus, we presume the Cl signal measured by XPS to originate from Parylene C. As the diX AM coating was thicker than the XPS sampling depth of 5 to 10 nm, we interpret the Cl signal as an indication that the diX AM coating was not completely closed. Concerning Oxygen content, amines are generally known to be prone to oxidation. St-Georges-Robillard [33] have recently investigated oxidation of diX AM on air and found simultaneous depletion of amino groups and increase of Oxygen content on a time scale of several days. The number of chemically available amino groups on the diX AM surface as determined by ESR Spectroscopy was 0.94 ± 0.33 1/nm^2^.

**Figure 2:**
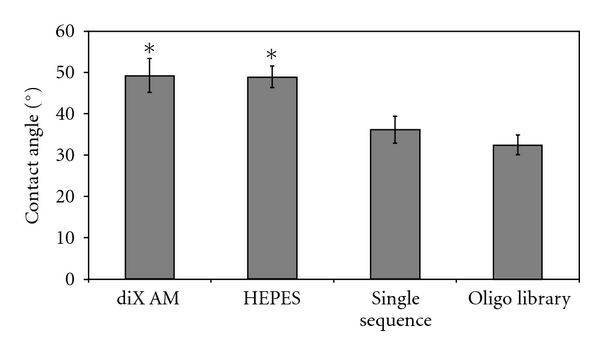
Contact angle measurements by the captive bubble method using PBS. Contact angle was significantly lower for oligonucleotide coated samples compared to diX AM or HEPES treated samples (*, *P* < 0.05, *n* = 6).

To examine if changed surface wettability has an impact on cell adhesion, samples were analyzed by the captive bubble method. Samples coated by a single oligonucleotide sequence or by the oligonucleotide library had significantly lower contact angles resulting in increased hydrophilic surface characteristics compared to diX AM (*P* < 0.001; Figure 2). This was independent of the used buffer (HEPES) as the contact angle for HEPES-treated samples did not differ from the angle of the diX AM surface.

### 3.2. Immobilization of Oligonucleotides and Shear Stress Resistance of Adhered Oligonucleotides

**Figure 3:**
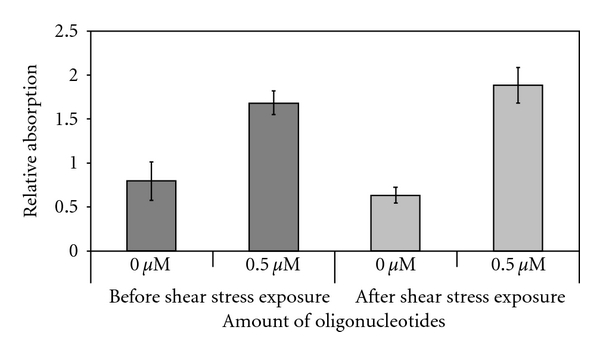
Oligonucleotide coating stability. Polypropylene sheets were coated with diX AM and oligonucleotides were applied to the coated surface. Oligonucleotides were detected by an ELISA system. Samples were exposed to a constant fluid flow inducing at least 9, 5 N/m^2^ shear stress on the surface for 1 h (*n* = 6).

On diX AM-coated surfaces, immobilized oligonucleotides were detected via fluorescence labeling of the oligonucleotides and an ELISA-System targeting the fluorescence label. After application of oligonucleotides, four washing steps were applied. Fluorescence and absorbance, respectively, were significantly higher, when oligonucleotides were applied to the diX AM surface (Figure 3, prior to shear stress exposure, data of fluorescence detection not shown). Immobilization by sole adhesion of the oligonucleotides on the diX AM-coated surface is possible. 

Oligonucleotides were exposed to physiologic shear stress to test if the adhesion is stable enough to withstand occurring shear stresses *in vivo*. For the assay, a mean wall shear stress of 9.5 N/m^2^ was chosen, as shear stress on native *in vivo* heart valve and artery surfaces are estimated to approximate 8 N/m^2^and 1.5 N/m^2^ [34, 35]. Accordingly, the chosen setup exceeded the maximum physiologic levels. The used fluid, Xanthan gum, simulated shear thinning viscosity behavior and particle characteristics of blood [36, 37]. No significant decrease of oligonucleotide amount on the sheared diX AM surface could be detected compared to the oligonucleotide control (*P* = 0.076; Figure 3).

### 3.3. Serum Stability of Adhered Oligonucleotides

**Figure 4:**
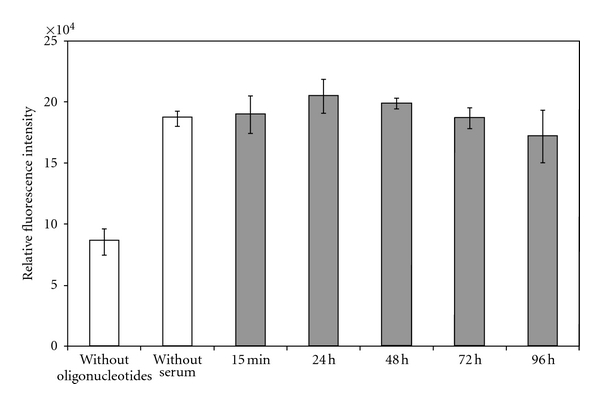
Stability against degradation by human blood serum of on diX AM-immobilized oligonucleotides. No significant degradation of oligonucleotides could be detected after 96 h incubation in comparison to the samples without serum incubation (*P* = 0.079, *n* = 9).

To exclude degradation of the applied oligonucelotides by human blood serum, samples were incubated between 15 min and 96 h with human blood serum. The mean values of each time point differed by ±9.6% from the mean of the sample without serum incubation (Figure 4). No significant degradation of oligonucleotides could be detected after 96 h incubation in comparison to the samples without serum incubation (*P* = 0.079).

### 3.4. Cell Adhesion and Viability on Oligonucleotide-Coated diX AM

**Figure 5:**
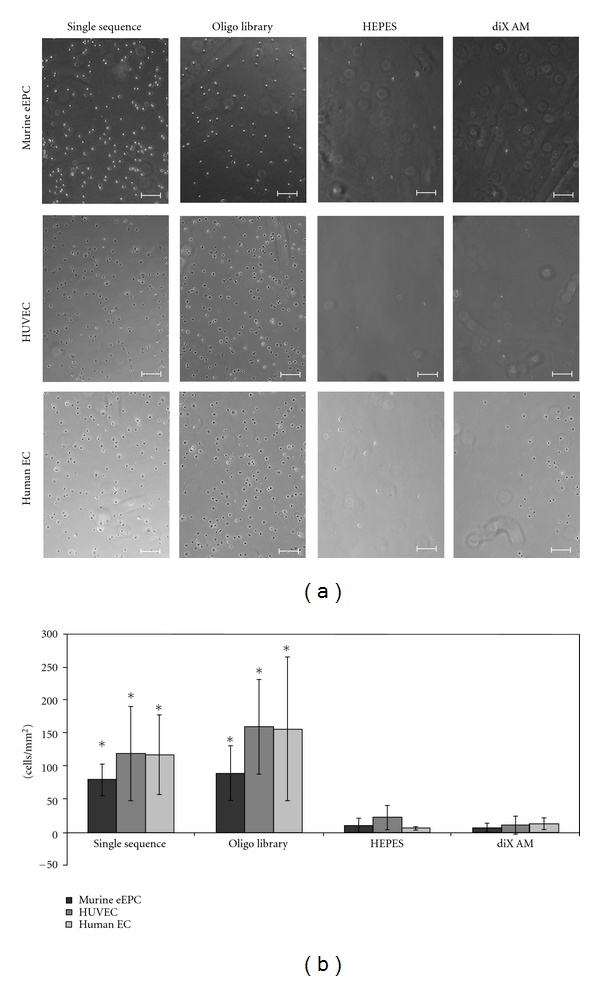
Cell adhesion to oligonucleotide-coated diX AM surfaces in FCS containing medium under dynamic conditions. Samples were incubated with 0,05 ∗ 10^6^ cells for 1 h in corresponding medium containing FCS. (a): Scale bar equals 200 *μ*m. (b): Number of adhered cells per mm^2^ was counted in microscopy images (*n* = 6). Groups marked with ∗ are significantly different to HEPES and diX AM samples (*P* < 0.05).

**Figure 6:**
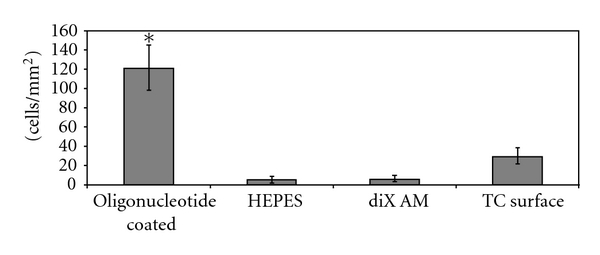
Adhesion of HUVECs to oligonucleotide-coated diX AM surfaces in FCS containing-medium under dynamic conditions compared to tissue culture (TC) surfaces. Samples were incubated with 0,05 ∗ 10^6^ cells for 1 h in medium containing FCS. Number of adhered cells per mm^2^ was counted in microscopy images (*n* = 9). Group marked with ∗ is significantly different to HEPES, diX AM, and TC surface samples (*P* < 0.05).

Cell adhesion experiments were conducted with different cell types in FCS supplied medium (10% for eEPC and human EC, 2% for HUVEC). DiX AM samples where oligonucleotides with the same sequence, an oligonucleotide library, HEPES, or nothing of all was applied were used. Cells of a murine embryonic EPC cell line (eEPCs) adhered predominantly to areas coated with either the single oligonucleotide sequence or the oligonucleotide library (Figure 5(a)). HUVECs and human EC derived from human saphenous veins showed the same behavior. In Figure 5(b), the mean number of cells per mm^2^ on each coating and cell type is shown. Cell number on oligonucleotide-coated surfaces was significantly higher than on uncoated diX AM or the HEPES control (*P* < 0.05). Thus, oligonucleotides enhanced the adhesion of all cell types compared to cell adhesion on diX AM or buffer-treated diX AM. Experiments (*n* = 9) containing controls on tissue culture (TC) surface were conducted with HUVECs. Cell adhesion on oligonucleotide-coated areas was significantly higher than on TC surfaces (*P* < 0.05), see Figure 6. 

**Figure 7:**
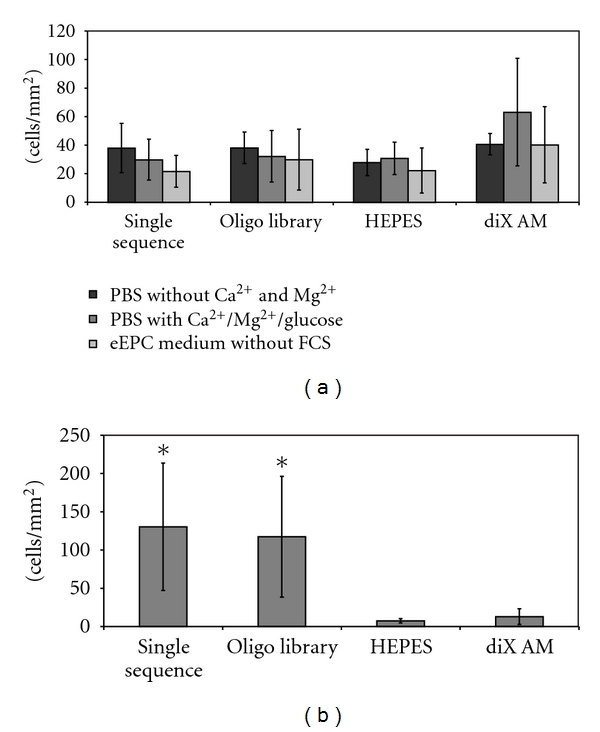
Influence of FCS on cell adhesion on oligonucleotide coated diX AM surfaces. (a) Samples were incubated with 0,05 ∗ 10^6^ eEPC cells for 2,5 h under dynamic conditions in medium or buffer without FCS. Cell number was counted in microscopy images (*n* = 6). ANOVA tests revealed no significant difference between the groups. (b) Samples were preincubated with eEPC Medium containing FCS for 2 h. Afterwards 0,05 ∗ 10^6^ eEPC cells were applied for 2,5 h under dynamic conditions in medium without FCS. Cell number was counted in microscopy images (*n* = 6). Groups marked with ∗ are significantly different to HEPES and diX AM samples (*P* < 0.05).

To examine the effect of the oligonucleotide coating on cell adhesion without previous protein deposition from FCS, eEPC cells were applied to the samples in serum-free medium or buffers. For cells applied to the samples in PBS without Ca^2+^/Mg^2+^, PBS with Ca^2+^/Mg^2+^/Glucose or serum-free eEPC medium no significantly enhanced adhesion to oligonucleotide-coated areas could be detected (Figure 7(a)). Cells adhered to all samples equally. To further investigate the effects of FCS diX AM and oligonucleotide-coated Petri-dishes were preincubated for two hours with FCS supplemented eEPC medium. Medium was removed and 5 × 10^4^ mouse EPC cells in serum-free medium applied for 2.5 h at 37°C, 5% CO_2_ on a three-dimensional orbital shaker. On Petri-dishes preincubated with FCS-containing medium and cells applied in serum-free medium, the same behavior of cell adhesion like in Petri-dishes where cells were applied in FCS containing medium was observed. Adhesion of cells was significantly higher on oligonucleotide-coated areas then on control areas (*P* < 0.05, Figure 7(b)).

**Figure 8:**
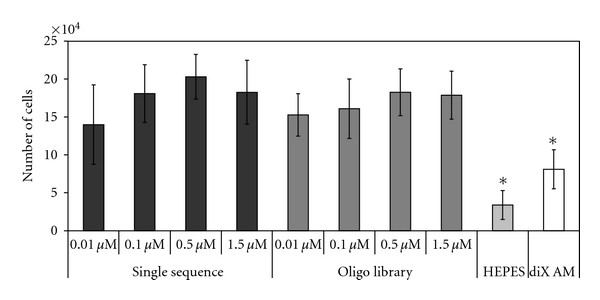
Incubation of eEPC cells on diX AM surfaces with different amounts of oligonucleotides. Amount of oligonucleotides did not significantly influence cell adhesion (*F*(7,16) = 1.598; *P* = 0,207). Groups marked with ∗ are significantly different to all oligonucleotide coated samples (*P* < 0.05, *n* = 3).

In a further experiment, it was investigated if the amount of oligonucleotide used for coating the diX AM surface plays a role in efficiency of cell adhesion. Different concentrations of the single oligonucleotide sequence and the oligonucleotide library were applied to a diX AM-coated 12-well plate. 5 × 10^4^ mouse EPC cells were applied to each well in FCS supplied medium and incubated at 37°C, 5%·CO_2_. After 1 h, all the nonadherent cells were washed off and the adherent cells incubated for a further 22 hours. After 22 hours, cell number was determined via the alamarBlue Assay. There was no significant difference between the samples with different oligonucleotide concentrations visible (Figure 8). Also, the utilization of a single sequence or of a library did not make any significant difference; but a significant difference in number of adhered cells was observed between oligonucleotide-coated samples and samples coated with diX AM only or on diX AM samples treated with buffer (HEPES) only (*P* < 0.05).

**Figure 9:**
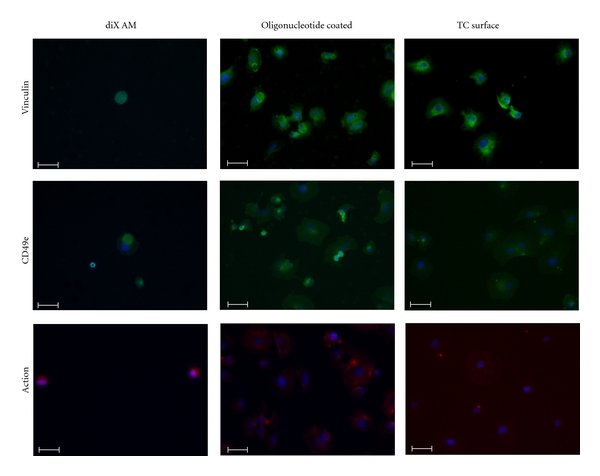
Staining for Vinculin, CD49e and F-Actin of HUVECs after 1 h hour incubation in FCS containing medium under dynamic conditions. Samples were incubated with 0,05 ∗ 10^6^ cells in medium containing FCS. Nonadherent cells were removed before staining. Scale bar equals 50 *μ*m. Nuclei are stained with DAPI (blue).

To further characterize cell adhesion, samples were incubated with HUVECs for 1 h and adherent cells were stained for Vinculin, CD49e (Integrin alpha 2 subunit) and F-Actin. Cells on oligonucleotide-coated surfaces showed a flat morphology and covered a bigger area than cells on diX AM control surfaces which showed a spherical morphology (Figure 9). Cell morphology on oligonucleotide-coated surfaces was similar to cell morphology on TC (tissue culture) surfaces. Cell density was higher on oligonucleotide-coated surfaces and on TC surfaces as on these surfaces more cells adhered than on diX AM control surfaces.

**Figure 10:**
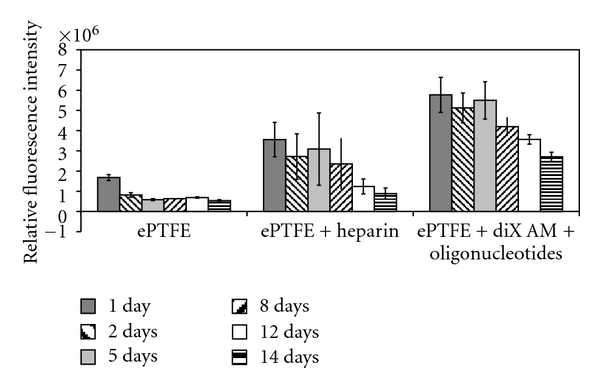
Viability of HUVECs on standard blood vessel graft materials (ePTFE and ePTFE coated with heparin) and on ePTFE coated with diX AM and oligonucleotides over a period of 14 days. Metabolic activity of cells was significantly higher for each time point on oligonucleotide-coated ePTFE compared to ePTFE or ePTFE with heparin (*P* < 0.05).

Viability of HUVECs over a period of 14 days was compared between two standard vascular prosthetic materials, ePTFE and ePTFE coated with heparin, and the newly developed coating, diX AM and oligonucleotides on ePTFE. Cell viability was significantly higher on oligonucleotide-coated ePTFE for all time points compared to ePTFE and ePTFE coated with heparin (Figure 10). 2.5 h after adding of cells, nonadherent cells were removed. Values for viability on day one after seeding are an indicator for cell adhesion promoting characteristics. Adhesion of HUVECs was significantly higher on oligonucleotide-coated ePTFE than on ePTFE or ePTFE with heparin. On all materials measured values for viability decreased over time. After 5 days, no viable cells could be detected on ePTFE any more. On ePTFE with heparin viability decreased on day 14 to 13 ± 10% of the value of day one. On ePTFE with oligonucleotides viability on day 14 was still 43 ± 5% of viability measured on day one.

### 3.5. Hemocompatibility

**Figure 11:**
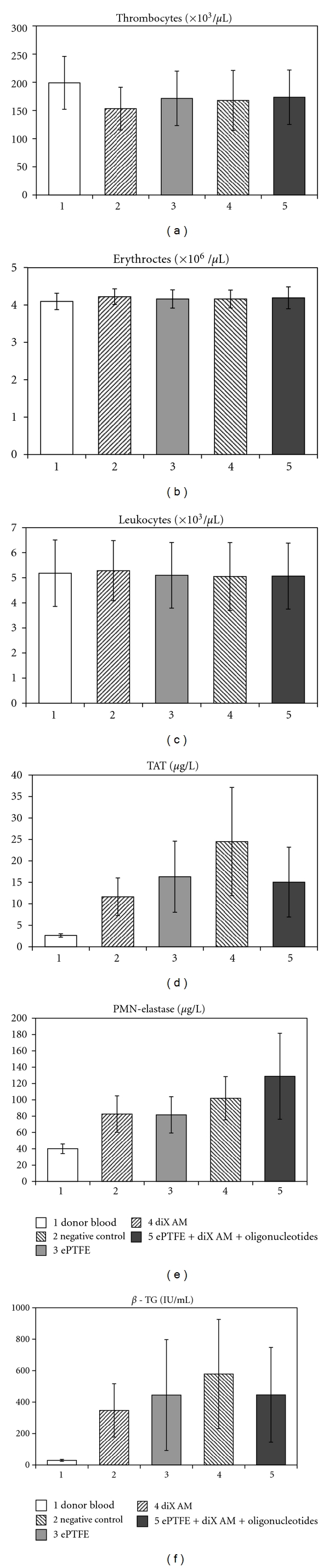
Hemocompatibility testing of oligonucleotide coated diX AM surfaces. Samples were incubated with human whole blood for 1 h at 37°C under continuous shaking. Cell number and different factors, which give information about the activation of thrombocytes (*β*-TG), granulocytes (PMN-Elastase) and the coagulation system (TAT) were measured in the supernatant. ANOVA tests revealed no significant difference between the groups (*P* > 0.05, *n* = 6).

Uncoated ePTFE, diX AM coated suspension cell culture plates and diX AM- and oligonucleotide-coated ePTFE samples were incubated with human blood for 1 h at 37°C on an orbital shaker. As negative control, wells of a suspension cell culture plate were used. The experiment was repeated with six different blood donors. To quantify cell adhesion platelets, erythrocytes and leucocytes were counted in blood samples following incubation. ANOVA tests revealed no significant decrease in number and therefore no significant adhesion to any of the samples (Figure 11). Surface-dependent activation of platelets and leukocytes was measured by determination of released *β*-thromboglobuline (*β*-TG) and PMN-elastase, respectively. *β*-TG is stored in the *α*-granules of platelets and released in large amounts after platelet activation. The release of polymorphonuclear- (PMN-)elastase takes place after activation of neutrophile granulocytes or collapse of these cells. It is broadly used to measure granulocyte activity during inflammatory response. Values for *β*-TG for oligonucleotide coated and uncoated dix AM surfaces did not differ significantly from negative control (*P* > 0.05). PMN-elastase was released to a higher extent by oligonucleotide-coated diX AM surfaces but there was no significant increase compared to the negative control (*P* > 0.05). Surface-dependent activation of the coagulation system was detected by measuring thrombin-antithrombin III (TAT). TAT is an inactive proteinase/inhibitor complex resulting from thrombin and its inhibitor antithrombin III. An increased TAT level is an indicator for thrombotic events. TAT level was not significantly increased for any of the samples (*P* > 0.05).

## 4. Discussion

Interfaces between implant surfaces and recipient tissue or blood play a crucial role for long-term performance and patency of cardiovascular implants. This study analyzed a novel oligonucleotide-coated diX AM layer in terms of coating stability, EC and EPC adhesion and viability under continuous shear stress application and hemocompatibility. These criteria were chosen as representative key characteristics for cardiovascular implants. Application of the coating concept for other implants is also conceivable.

The oligonucleotide coating showed excellent adhesion properties for human EC and murine EPC under continuous shear stress application as well as good viability properties over a period of 14 days for HUVECs. Cell morphology of HUVECs after adhesion on oligonucleotide-coated surfaces was similar to cell morphology of cells adhered to tissue culture surfaces. This qualifies the material for *in vivo* endothelialization either by EC migration from adjacent native tissue or by EPC capture and adhesion from the blood stream. Stability of the oligonucleotides coated on the diX AM layer was excellent. The applied oligonucleotides withstand shear stress in the amount of shear stress occurring in blood vessels *in vivo*. Additionally, the oligonucleotides were not degraded by human blood serum during prolonged exposure of 96 hours. Maintained hemocompatibility was confirmed by testing for thrombocyte, granulocyte, and coagulation system activation.

A combination of two parylenes, with an intermediate layer of Parylene C and the outer layer of diX AM, an amino-modified parylene derivate, was chosen as a protection film between synthetic material and tissue or blood, as polymers of the parylene family are known to offer good elasticity, biostability and biocompatibility and can be coated easily to different substrate materials even of irregular shape. Parylene C coatings are currently used as protection films for biomedical devices, for example, metal stents, implantable electrode probes, and electronic circuitries [25, 26, 38]. The limitations of Parylene C for cardiovascular and other tissue engineering approaches are the low cell adhesion characteristics [27, 39]. Other parylene derivates such as diX AM offer better growth capacities [39]. Even so we observed low adhesion rates of cells to diX AM-coated areas. Additional coating of diX AM with oligonucleotides enhanced cell adhesion significantly and allowed stable and confluent growth of the cells.

These oligonucleotides were robustly immobilized on the diX AM surface. Shear stress up to 9,5 N/m^2^ was applied to the samples, which exceeded the maximum shear stresses observed in blood vessels and heart valves [34, 35, 40]. Xanthan gum was used in the shear stress test fluid to simulate rheological properties of blood [36, 37]. Shear stress stability is crucial as denuded implants exhibit highly thrombotic surface characteristics. Also, DNA molecules may be easily degraded and their solubility in aqueous solutions is high [19]. This was not observed in the present study. After immobilization on diX AM, the oligonucleotides were retained in both the aqueous solutions used in the shear experiments and during incubation with human blood serum.

Interestingly, it did not matter whether a single sequence or an entire oligonucleotide library was used, both enhanced cell adhesion equally. The mechanism of enhanced attachment, therefore, is more likely to be dependent on the overall characteristics of DNA-oligonucleotides than on a specific sequence. As cells were only able to adhere, when they were added to the oligonucleotide-coated surface in FCS supplied medium or when the coated samples were preincubated in medium containing FCS, the mechanism of enhanced cell attachment maybe mediated through attachment-facilitating serum proteins deposited on the surface from FCS. It has been reported in the literature that deposition of serum proteins like fibronectin and vitronectin contained in serum-supplemented culture medium on cell culture material surfaces mediates initial cell attachment, spreading, and behavior [41, 42]. No enhanced cell adhesion could be detected for serum-free medium or PBS. Even supplementation of PBS with calcium, which plays a major role in adhesion via cadherins [43, 44] did not favor cell adhesion. Oligonucleotide coating obviously changes surface properties in a way that facilitates adsorption of functional proteins. A factor that was shown to affect protein adsorption is surface wettability. Protein adsorption is usually higher on hydrophobic surfaces [45, 46], but protein function is presumably maintained better after adhesion to hydrophilic substrates [47–51]. The oligonucleotide coating changes surface wettability to a more hydrophilic condition compared to the diX AM coating as shown by contact angle measurements. This corresponds to the observed enhanced cell adhesion. Therefore, the more hydrophilic oligonucleotide surface may be involved in this phenomenon, but other determinants such as surface charge might also play a role in cell attachment and hemocompatibility. The ability of materials to adsorb proteins (in an active state) from serum determines their ability to support cell adhesion and spreading [52] and, hence, is an important aspect of their biocompatibility. It will be very interesting to further characterize this adsorption in a future work.

Viability of the adhered cells was measured over a time period of 14 days. As a high number of cells were used for seeding, samples were assumed to be confluent on day one. Viability on oligonucleotide-coated samples decreased to 43 ± 5% on day 14. As the alamarBlue assays measure metabolic turnover, this might be due to less metabolic activity as further cell proliferation might not have been possible. Measured viability on ePTFE and ePTFE with heparin decreased more dramatically. In these cases, cell loss is probable. ePTFE coated with diX AM and oligonucleotides, therefore, showed considerably improved properties for cell growth. Additionally, the similar cell morphology of HUVECs adhered on oligonucleotide-coated surfaces like on tissue culture surfaces as shown by staining for Vinculin, CD49e, and F-Actin confirms the good properties for endothelial cell growth of oligonucleotide-coated surfaces.

For blood contacting material, hemocompatibility is a further important point. Many surface modifications used for engineering of material interfaces for EC adhesion that bind EC also will bind platelets and initiate thrombosis. Here, we provide data, demonstrating that the adhesion of erythrocytes, thrombocytes, and leucocytes was low to the oligonucleotide-coated ePTFE. This is an indication that oligonucleotide-coated diX AM surfaces do not activate inflammatory or thrombogenic responses. The measured values confirmed this result by not showing any activation of thrombocytes (*β*-TG), granulocytes (PMN-elastase) or the coagulation system (TAT) by oligonucleotide-coated ePTFE. Values for all examined variables were in the same range as the values for clinically routinely used standard ePTFE material (Gore-Tex Cardiovascular Patch, W.L. Gore & Associates, Inc., Flagstaff, Arizona, USA). Furthermore, the determined values were in a similar range as known values for materials with excellent hemocompatibility previously published: *β*-TG ranges around 500 IU/mL for biopassive or active coated membrane oxygenators (Jostra Quadrox, Maquet Cardiopulmonary, Hirrlingen, Germany) [53], glutaraldehyde-fixed pericardium [54] or star-PEG-modified substrates [55]. Values for PMN-elastase in literature range between 300–400 *μ*g/L for coated membrane oxygenators [53, 56]. Data for TAT revealed levels of 6–8 *μ*g/L for star-PEG-coated surfaces [55] and nitinol stents [57]. Positive controls are known to range between 2300–6000 IU/mL for *β*-TG [53–55], 450–1000 *μ*g/L for PMN-elastase [53, 56], and 500–6000 *μ*g/L for TAT [55, 57]. Other studies have reported nonimmunogenic characteristics of oligonucleotides [15, 17, 18]. This might be due to their small size and their natural occurrence in the human body thus being no foreign body material [15, 16]. Advantages of oligonucleotides as coating material are easy synthetization and many functional groups for coupling of additional molecules, such as growth factors. The amino groups of the diX AM surface can also be utilized for molecule coupling. We determined an amount of free amino groups on diX AM polymer coating of about 1 million per *μ*m^2^. To confirm the applicability of the presented DNA coating for *in vivo* endothelialization of cardiovascular implants, further experiments to clarify the adhesion properties of EC and EPC in human blood vessel environment conditions are required. As diX AM can be coated to substrates of various shapes and material, the coating process can be easily transferred to currently used vessel graft materials. In this work, we developed the successful coating of ePTFE with diX AM and oligonucleotides by surface activation using microwave plasma processing and subsequent chemical vapour deposition of diX AM and oligonucleotide adsorption.

This study revealed DNA-oligonucleotides coated to diX AM surfaces as attractive coating materials for cardiovascular implants. They are shear resistant, nondegradable by human blood serum, offer excellent adhesion properties for EC, and are hemocompatible. Furthermore, they might support adhesion of circulating EPC or transanastomotic ingrowth of EC. This opens new opportunities for the manufacturing of “off-the-shelf” cardiovascular implants to achieve *in vivo* endothelialization after implantation. Additionally, immobilization of oligonucleotides on other types of implants, for example, orthopedic tissues might enhance their incorporation into surrounding tissues.
